# Social Media Discourse Related to Caregiving for Older Adults Living With Alzheimer Disease and Related Dementias: Computational and Qualitative Study

**DOI:** 10.2196/59294

**Published:** 2024-06-19

**Authors:** Andrew C Pickett, Danny Valdez, Kelsey L Sinclair, Wesley J Kochell, Boone Fowler, Nicole E Werner

**Affiliations:** 1 Department of Health & Wellness Design School of Public Health Indiana University Bloomington, IN United States; 2 Department of Applied Health Science School of Public Health Indiana University Bloomington, IN United States

**Keywords:** caregiving, dementia, social support, social media, Reddit

## Abstract

**Background:**

In the United States, caregivers of people living with Alzheimer disease and Alzheimer disease–related dementias (AD/ADRD) provide >16 billion hours of unpaid care annually. These caregivers experience high levels of stress and burden related to the challenges associated with providing care. Social media is an emerging space for individuals to seek various forms of support.

**Objective:**

We aimed to explore the primary topics of conversation on the social media site Reddit related to AD/ADRD. We then aimed to explore these topics in depth, specifically examining elements of social support and behavioral symptomology discussed by users.

**Methods:**

We first generated an unsupervised topic model from 6563 posts made to 2 dementia-specific subreddit forums (r/Alzheimers and r/dementia). Then, we conducted a manual qualitative content analysis of a random subset of these data to further explore salient themes in the corpus.

**Results:**

The topic model with the highest overall coherence score (0.38) included 10 topics, including caregiver burden, anxiety, support-seeking, and AD/ADRD behavioral symptomology. Qualitative analyses provided added context, wherein users sought emotional and informational support for many aspects of the care experience, including assistance in making key care-related decisions. Users expressed challenging and complex emotions on Reddit, which may be taboo to express in person.

**Conclusions:**

Reddit users seek many different forms of support, including emotional and specific informational support, from others on the internet. Users expressed a variety of concerns, challenges, and behavioral symptoms to manage as part of the care experience. The unique (ie, anonymous and moderated) nature of the forum allowed for a safe space to express emotions free from documented caregiver stigma. Additional support structures are needed to assist caregivers of people living with AD/ADRD.

## Introduction

### Background

There are an estimated 11 million people in the United States currently living with Alzheimer disease and Alzheimer disease–related dementias (AD/ADRD)—a number that is expected to more than double by 2050 [1]. Most people living with AD/ADRD receive informal (ie, unpaid) care support from family members or other personal connections due to the high costs of professional care, the limited capacity of assisted living and memory care facilities, and a strong desire to age in place [2-5]. Recent estimates suggest informal AD/ADRD caregivers in the United States provide >16 billion hours of care labor annually. This has led to calls from national health organizations to prioritize the expansion of support systems for informal caregivers of people living with AD/ADRD. Caregiving for people living with AD/ADRD is a complex and challenging role that is associated with a broad set of suboptimal economic, physical, and psychological outcomes. For example, caregiver burden is associated with negative mental health outcomes, including high levels of stress and depressive symptomology. However, social support may offer a means for reducing caregiver burden and improving health outcomes for both caregiver and care recipient. This study aimed to understand the ways AD/ADRD caregivers seek and experience social support on the web, specifically through the social media platform Reddit (Reddit, Inc).

### Caregiving for Individuals Living With AD/ADRD

Recent studies have estimated that approximately 15 million Americans currently provide care for individuals living with AD/ADRD [6]. Most are classified as informal caregivers, meaning they are not paid for labor and time associated with care responsibilities and are typically family members or friends of the person with AD/ADRD. The average life expectancy for individuals diagnosed with dementia can vary widely, from 3 to 10 years, depending on the specific diagnosis, age at the time of diagnosis, and other health factors [7]. Not surprisingly, the caregiving experience can also vary widely. A recent study found that near the time of diagnosis, people with AD/ADRD received an average of 151 hours (approximately 6.5 days) of caregiving monthly, typically provided by 1 caregiver. However, over time, their needs progress, requiring nearly twice the hours of care and support of additional caregivers [8].

Caregiver burden describes the multifaceted strain faced by individuals in providing care, which may include financial, emotional, and physical stressors [9,10]. The challenges of AD/ADRD care are associated with significant health impacts for caregivers [11,12]. Providing care is often associated with higher levels of reported stress [13]. Furthermore, caregivers report high levels of psychiatric symptoms, most commonly depressive symptoms—particularly as the care recipient’s AD/ADRD symptomology progresses [14,15]. Caregivers also report high levels of financial strain related to high costs of care and lost earning potential due to time commitments of care responsibilities [9,16].

Regardless of the dementia subtype, a broad range of neuropsychiatric symptoms, including both behavioral and cognitive changes, can present [17]. Behavioral symptoms of AD/ADRD, for example, can include confusion, aggression, and increased hospital or emergency department visits. Dementia is also associated with neuropsychiatric symptoms, including agitation, depression, hallucinations, anxiety, and apathy [18-20]. Furthermore, dementia is often associated with reduced function of other organs, resulting in symptoms such as voice or speaking challenges, skin injuries, urinary incontinence, constipation, urinary tract infections, dental and vision problems, and hearing loss, among others [18-20]. Some symptoms, such as anxiety and depression, are fairly common across patients with AD/ADRD and with disease progression [20]. Others, such as psychosis, aggression, and agitation, are often exacerbated as cognitive decline increases and are thus associated with steep increases in caregiver burden due to the impact these symptoms have on the completion of basic activities of daily living [18].

As AD/ADRD progresses, more severe symptoms reduce individuals’ capacity to independently complete activities of daily living, thereby necessitating increased assistance and caregiver supervision [21]. Therefore, the time spent on caregiving activities and the number of individuals providing care generally increase across the disease progression [2,3]. Naturally, increased time spent on care, combined with increasingly severe symptoms to manage, is associated with increased feelings of burden. As such, caregivers often spend less time and energy on their own self-care, particularly as the care recipient’s neuropsychiatric symptoms worsen [22]. The emotional, financial, and physical strain of caregiving may contribute to reduced overall health of caregivers [5]. AD/ADRD caregivers often report high levels of stress and depression themselves, may get poor sleep, and neglect their own well-being (eg, diet and physical activity) [11,14,15]. As such, recent epidemiological research has found spousal caregivers are at increased risk of dementia themselves and has found caregiving to be an independent risk factor for mortality [23,24]. Notably, strategies and interventions are needed to reduce burden and improve outcomes for both the caregiver and care recipient.

### Social Media and Caregiver Social Support

Over the past 3 decades, people have increasingly sought health information and support on the web. In high-resource countries, more than half of the adults use the internet for health reasons, often searching for information related to symptomology, diagnoses, and treatment options for health conditions. Online health information seeking is particularly common when users themselves or a close family member have a chronic health condition. While there are numerous websites from which users may access health information, they are increasingly likely to do so specifically via social media platforms.

Defining and delineating what constitutes a “social media platform” has proved challenging; however, most definitions broadly include a limitation to digital technologies, content generation by users, and the capacity for users to interact or share directly with others [25-27]. Over time, the use of social media has exploded, with a vast majority of adults in the United States reporting regular engagement with at least 1 platform and an average daily use of >2 hours [28,29]. Important to the current research, social media is widely used as a tool for people to connect with mutual friends, interests, circumstances, and hobbies [30-32].

To date, research related to caregivers’ use of social media has spanned many chronic conditions (eg, cancer, diabetes, and physical and mental disability); ages (ie, older adults and children); and platforms (eg, Twitter, X, Facebook, Instagram, and Reddit). Much of this exploratory work has examined use patterns and user needs [33,34], broadly suggesting caregivers most often use social media to exchange information related to care recipient health, psychosocial issues, and daily care activities [33-35]. Among the latter, caregivers may use social media to discuss activities of living, sleep, diet, finance, showering or bathing, transportation, medical care, and formal disease diagnoses [35]. Some research suggests online support is associated with a positive impact on the emotional well-being of medical caregivers, especially for those who used online support for a long term [36]. However, to date, limited literature exists examining the use of social media for support-seeking among AD/ADRD caregivers, specifically [35,36].

AD/ADRD caregivers may be particularly likely to seek support on the web due to myriad factors that limit social support in their offline lives and community settings. For example, AD/ADRD caregivers face documented stigmatization and progressive social isolation as care recipient symptomology worsens and it becomes more challenging to engage in public social settings [6,34,37]. As such, early social interventions for AD/ADRD caregivers have often focused on creating shared spaces with others in similar situations [5,38]. However, rural residents and others who are not located near needed resources (eg, respite or adult day care and support groups) may face challenges in finding others who have similar experiences or a shared understanding of care responsibilities [39]. Furthermore, AD/ADRD caregiving requires substantial time and financial commitments, which create logistical limitations on opportunities for social engagement outside of one’s care responsibilities [23,34,40]. Finally, even in shared spaces with similar others, it may be challenging to express complex or difficult emotions that are common among caregivers [15,22]. Notably, AD/ADRD caregivers may be particularly likely to pursue support on the web via social media, rather than through in-person or community-based programs.

Therefore, this study aimed to explore how caregivers use the social media platform Reddit, with particular emphasis on social support and AD/ADRD information seeking. To this end, we first collected and applied computational natural language processing (NLP) tools to a large corpus of Reddit posts to identify salient themes across the site. We then conducted a manual qualitative content analysis of a random subset (ie, 657/6563, 10.05%) of the corpus to gain a more nuanced understanding of support and information-seeking behaviors of AD/ADRD caregivers through the site.

## Methods

### Ethical Considerations

For this project, we analyzed only existing data (ie, publicly available information), posted directly to Reddit. All study procedures were approved by the Indiana University Institutional Review Board (#23662).

### Data Collection

We collected data for this study from Reddit, a social news aggregation, content rating, and discussion social media platform. Unlike other social media platforms, including Instagram, TikTok, and X (formerly Twitter), Reddit represents a forum of communities where people can “opt in” to subreddits, which are moderated subspaces within the wider platform, dedicated to a certain topic. This allows users to seek community and interaction based on specific interests or needs [41]. Subreddit content varies extensively. However, in recent years, subreddits specific to health and well-being–related topics have become increasingly used by people in need of social support, connection, or solidarity [42,43]. Reddit data are also unique in that posts are unincumbered by length limitations. Therefore, data collected from Reddit tend to be more comprehensive and nuanced compared with data from other social media platforms.

Using the Python Reddit API Wrapper [44], a third-party Reddit data scraper, we identified 2 subreddits specific to our research purpose (r/Alzheimers and r/dementia). We then programmed the Python Reddit API Wrapper to collect new, popular, and trending posts, along with relevant metadata, in both subreddits between May and June 2022. These data were saved as a single CSV file for further computational and qualitative data analysis. Upon completion of data collection and excluding duplicates, blank entries, and those deemed irrelevant to discussions about AD/ADRD, we retained 6593 posts.

### Analysis

**Figure 1:**
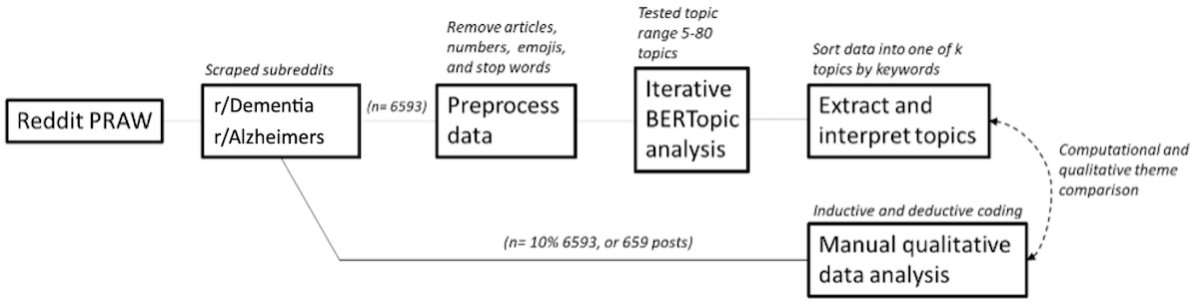
Workflow depicting computational and qualitative data analyses pipeline. PRAW: Python Reddit API Wrapper.

Given our exploratory research purpose, we undertook a two-step process to analyze our data: (1) computational analyses and (2) manual qualitative coding and review. This 2-step approach afforded the ability to identify topics embedded across the entirety of our data using NLP methods and to understand the meaning of each topic at a deeper and more nuanced level through traditional qualitative analyses (Figure 1). We offer a brief explanation of each method applied to the data in subsequent sections.

### Natural Language Processing

#### BERTopic

Topic models refer to any series of NLP tasks that consolidate large language data sets into representative topics or themes. While there are a variety of topic modeling tools, including the highly established latent Dirichlet allocation (LDA) approach, our study used a neural network pipeline leveraging Bidirectional Encoder Representations from Transformers (BERT) vectors using the BERTopic topic modeling tool [45,46]. Indeed, BERTopic is unique among established LDA approaches in that this pipeline specifically uses neural networks and BERT embeddings to approximate latent topics. Topics derived using the BERTopic tool tend to be clearer and more nuanced due to the encoding process, where raw text data are compared against a large language data set. At this stage, raw text data are converted into numerical form, which creates dense vector representations for each document in a given data set. These representations capture contextual nuances of each word across documents, which is not attainable using LDA or other probabilistic topic modeling approaches. Vectors are calculated for each document. As these vectors are difficult to model and interpret, we then perform a dimensionality reduction technique, principal components analysis. Following dimensionality reduction, we extract a series of topics that represent a synopsis of the entire corpus.

#### Coherence Score Check

BERTopic, and most other topic modeling tools, can generate any number of latent topics specified by a predetermined parameter *k*. However, this fixed topic number may not always reflect the optimal number of topics for a given corpus. Therefore, multiple models may be run and compared, retaining the best overall topic solution for the research. Coherence scores, which are a statistical value predicting the overall interpretability of topics, are a common metric for assessing model fit and choosing the optimal solution. Coherence scores are calculated using pointwise mutual information, which is a measure of the strength of association between 2 words in each document and a collection of documents. In brief, this calculation tells us the degree to which 2 words are more likely to appear together than what would be expected by chance. Therefore, higher coherence scores generally indicate a more interpretable topic solution, as words that co-occur in the corpus more often than they would by random chance are sorted together as topics.

### Qualitative Content Analysis

To support findings derived from computational analyses and to better understand nuance related to social support and AD/ADRD information seeking within our data, we conducted a manual qualitative content analysis of a subset of posts previously analyzed. For this analysis, we adopted an approach that was both inductive and deductive in nature. Given our specific research interests related to the ways users sought support on the web, we deductively mapped data codes onto existing conceptual frameworks related to social support and known AD/ADRD behavioral symptomology. However, we inductively allowed for additional codes to emerge from the data.

### Procedure

#### Natural Language Processing

Once we completed data collection, we began preprocessing our data, a common procedure in computational analyses that seeks to standardize and add cohesive structure to messy text data. Part of this standardization includes removing parts of speech that may detract from the clarity of the models, including first-person names, numbers, special characters, emojis, articles, and a series of stop words. Once our data were preprocessed, we proceeded to analyze the data with the BERTopic tool. To identify the optimal number of topics, we performed an iterative topic model analysis that tests a range of topics by iterations of 5 (eg, 5, 10, 15,*......k).* For each iteration, we calculated coherence scores. We selected the optimal number of topics based on the highest coherence score among topics, ranging from 5 to 80. Once we identified the optimal topic number, we performed a sorting function, which triaged all tokenized sentences into one of *k* latent topics. This sorting feature allowed us to examine topic numbers, keywords associated with each topic, and the number of parsed sentences sorted in each topic. Once computational analyses were complete, we performed a manual review of a random 10.05% (657/6563) of posts within our larger sample. These posts were manually coded and independently compared to the BERTopic output to ascertain overlap.

#### Qualitative Content Analysis

Initial coding was undertaken by 3 individuals on the research team. First, coders trained together on sample data that were not included in later analyses. This initial coding was subsequently reviewed by 2 established researchers who were not initial coders. After review, the research team met 3 times to review the coding of the training data, which helped crystallize code definitions and identify early emergent themes. Any disagreements in the coding of these training data were discussed until a consensus was reached.

After training, a similar coding process was adopted for the larger data set. Initial coding was completed by the same 3 individuals who provided initial open codes in the training data. This initial coding was then reviewed independently by 2 established researchers from the team for consistency and accuracy. We began to draw connections between initial open codes via axial and selective coding processes to arrive at the final structure, as outlined in the Results section. The coding team met twice to discuss the overall coding structure, again resolving disagreements via discussion until a consensus was reached.

## Results

### Unsupervised Topic Modeling

Our first set of analyses used NLP to identify the broad themes discussed by users in subreddits related to AD/ADRD. The optimal solution, as determined by the coherence score, included 10 topics, with a coherence score of 0.38. Table 1 provides a summary of each topic, including each topic name, keywords associated with each topic, and its proportional representation within the larger corpus. Importantly, our analysis tokenized data at the sentence level. We chose to analyze at the sentence level, rather than the post level, to identify more granular topics within the corpus. Had we analyzed at the post level, the algorithm would have sought to identify the primary overarching theme across the entire post. Because Reddit posts have no limitations on length, users may include multiple topics in a single entry; such intrapost thematic variability would be poorly reflected with a postlevel analysis, likely resulting in very general and hard-to-interpret topics. Therefore, to avoid such a loss of nuance, we chose the more granular sentence-level approach.

Proportionally, the topic with the greatest representation in our corpus was topic 0, “Reddit inquiries, narratives, and advice,” comprising 17.45% (16,152/ 92,562) of sentences in the total corpus. Other prominent topics include topic 1, “venting/expressing feelings and emotions” (12,652/ 92,562, 13.67%); topic 2, “moving- and housing-related adjustments” (12,090/ 92,562, 13.06%); and topic 3, “ADRD diagnosis and other acute illnesses” (10,695/ 92,562, 11.55%). The remaining topics were marginally consistent in terms of distribution, with topic 9, “hospital, assisted living, and memory care,” having the lowest representation of 5.4% (4994/92,562).

Topics derived using any topic modeling tool are potentially overly generalized or difficult to parse if topics share similar keywords. As such, additional review of the topic solution by researchers with topical knowledge is needed to ascribe meaning to each latent topic. To do so, we applied a sorting function based on keywords to sort tokenized sentences into 1 of 10 possible topics. We then reviewed individual posts to understand and contextualize each topic. Table 2 provides excerpts of posts sorted into each topic to assist the reader in understanding the latent topics identified in the corpus. We observed some generalized themes, as well as more specific AD/ADRD caregiving and contextual topics.

The first 2 identified topics are more general in nature. For example, topic 0, “Reddit inquiries, narratives, and advice,” is quite general and reflects the common language of Redditors asking questions to the community. What follows in any given sentence varies (eg, diagnoses, caregiving advice, or difficulties associated with AD/ADRD symptoms), but there was a common theme of seeking, whether related to information or emotional support. Similarly, topic 1, the second most common in the corpus, encapsulated users’ many and varied expressions of emotion. These more generalized themes are often found in the first few sentences of a Reddit post, wherein the user expresses a wider sentiment before providing more specific and varied details.

Another grouping of topics related to the varied contexts of AD/ADRD caregiving and related decision-making. Topic 2, for example, focused on housing- and moving-related concerns, where users often sought information and advice related to the appropriate time to limit a loved one’s capacity to live alone or strategies for ensuring the safety of community-dwelling individuals living with AD/ADRD. Topics 7 and 9, respectively, centered on more professional care settings. Topic 9 focused on hospitals, assisted living, memory care, or other such facilities; many of these posts were regarding providing or seeking information about proper care, identifying a quality facility, financing options, or the appropriate timing for seeking professional care support. Finally, topic 7 included posts related to hospice (ie, end-of-life) care. Notably, users often sought guidance on the appropriate time to move their loved ones to this type of care and emotional support at this challenging stage.

The remaining topics related to different aspects of the caregiving experience, including the impact of diagnosis (and comorbidities) and care on the family, caregiver burden, and 2 general caregiving topics. In topic 3, for example, we observed many posts that related to physical symptoms related to both dementia and related health challenges (eg, injuries from falls). For many who may not have opportunities to connect with similar others, platforms such as Reddit may be the only source of support available (eg, “I really just don’t know where else to turn”). Others either sought or provided specific advice related to managing certain symptoms (eg, aggression and anxiety) and strategies for managing the burdens of care.

**Figure 2:**
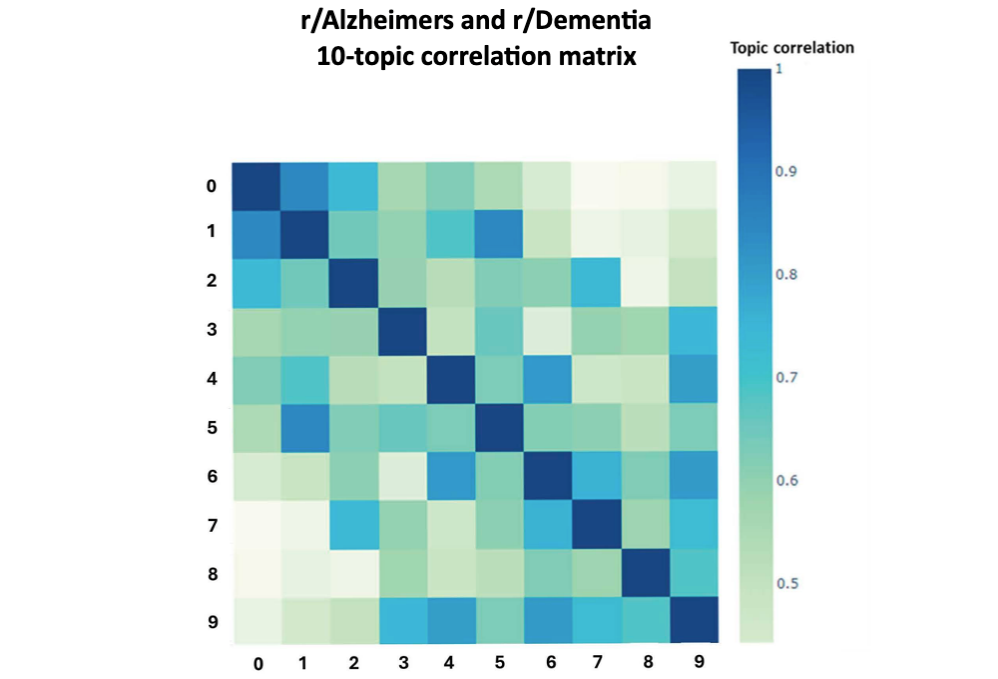
A 10-topic solution correlation matrix denoting topic similarity.

Notably, topics 6 and 4 were highly interrelated and discussed a broad set of caregiving-related topics. While the content in these 2 topics was highly related and posts were often similar, topic 4 focused more specifically on women, while topic 6 was less-based on gender overall. Because of the unsupervised nature of NLP, wherein latent topics are generated based on word co-occurrence but are not constrained to be perfectly orthogonal, it is possible to generate highly correlated topics, as in our data. To further contextualize this overlap (and other bivariate relationships between topics), we generated a correlation matrix using the BERTopic Python module (Figure 2). This figure offers a visual representation of relative topic overlap, with darker colors indicating higher levels of overlap. Not surprisingly, we can see high levels of overlap between topics 6 and 4. The presence of overlapping or highly correlated topics may imply the existence of more generalized themes or a hierarchical structure, which is beyond the scope of this analysis.

**Table 1:** BERTopic 10-topic solution, including topic name, top associated words, and overall corpus representation (N=92,562).

Topic ID	Topic name	Short name	Top associated words	Sentences, n (%)
0	Reddit inquiries, narratives, and advice	Inquiries	ago, today, night, say, asked, ask, day, asking, guess, idea	16,152 (17.45)
1	Venting or expressing feelings and emotions	Expressing emotion	situation, feel, say, talk, feeling, felt, saying, talking, thinking, think	12,652 (13.67)
2	Moving- and housing-related adjustments	Housing or moving	moved, house, moving, apartment, bedroom, phone, home, stay, job, contact	12,090 (13.06)
3	AD/ADRD^a^ diagnosis and other acute illnesses	AD/ADRD diagnosis	treatment, patient, med, dr, doctor, hospital, diagnosis, medical, appointment, medicine	10,695 (11.55)
4	Women and caregiving	Women and caregiving	mom, mother, mum, parent, shes, upset, daughter, aunt, angry, grandma	9065 (9.79)
5	AD/ADRD caregiving burden	Caregiver burden	depression, depressed, cope, stress, stressed, caring, grief, feel, burden, family	7637 (8.25)
6	Family caregiving	Family caregiving	grandparent, grandchild, sibling, parent, caregiving, grandmother, grandpa, grandma, family, aunt	7624 (8.24)
7	Hospice care and decision-making	Hospice	caregiving, caregiver, hospice, wheelchair, hospital, aide, health care, rehab, nursing, home	6280 (6.78)
8	AD/ADRD impact	AD/ADRD impact	dementia, alzheimer, alzheimers, elderly, diagnosed, grandma, grandmother, diagnosis, grandpa, impairment	5373 (5.80)
9	Hospital, assisted living, and memory care	Professional care settings	Hospital, hospice, grandmother, nurse, nursing, grandma, illness, diagnosis, caregiver, medical	4994 (5.40)

^a^AD/ADRD: Alzheimer disease and Alzheimer disease–related dementias.

**Table 2:** Representative post excerpts per topic, derived from our sorting function.

Topic ID	Topic name	Representative quotation
0	Reddit inquiries, narratives, and advice	“Just curious if anyone has more information.” “I'll probably poke around this subreddit every once in a while.” “Hey Lovelies - just bouncing in to share a little tip that has made a HUGE difference in our household.
1	Venting or expressing feelings and emotions	“I made the mistake of lashing out when he asked why I didn't do his stretches Monday morning, saying ‘do you really think I have time in the mornings to do that?’...I feel like he only wants me around to do everything for him” “Like, I have no one to talk to. No one will understand. I just want to run away and be left alone. I did not ask for this to be my life” “Even before the pandemic I felt like my soul was being pulled from me. I just wish I could be around ‘normal’ people”
2	Moving- and housing-related adjustments	“We recently bought some cameras and a door sensor and suddenly we know that he’ll go outside at 3 AM for whatever reason. Are there locks that can be connected to a C02 or smoke alarm?” “He also shows no interest in leaving his home; he spends his days in his recliner reading or watching TV.” “I want her to go to AL, which in our area averages about $5k a month [for care]. She has enough money in the bank to live in AL for 4-5 years. She doesn't want to go to AL because ‘It’s too expensive.’”
3	AD/ADRD^a^ diagnosis and other acute illnesses	“They told us they could force-feed her via intravenously or put a tube down her throat or stomach. The doctor’s other suggestion was to ‘make her comfortable.’” “And then one morning...she fell and broke her hip because she didn't use her walker while heading to the bathroom.” “Symptoms started last November. By January she was hospitalized. After the diagnosis of LBD, medical practitioners started treating her differently.”
4	Women and caregiving	“I picked out a room yesterday and while the guilt is getting to me too, my sibling is having a harder time dealing with it. My heart is breaking over this cat and the bond my mom has with him.” “We gave our youngest kids OUR house and animal because I had made a promise to my mother she wouldn't go into a home years before this s**t got real. My mother was still in total denial, and we were basically ignorant in a not stupid way.” “My grandma is 75 years old and has been acting kinda not herself most of the time since February. But by this time I’ve noticed that she’s often confused, asks the same stuff a lot sometimes, has spontaneous bad mood, crying thinking of something bad.”
5	AD/ADRD caregiving burden	“My thoughts run to upping my anxiety meds, cry in my mothers arms, post vague rants on Facebook, depress friends with the tearful story about my situation or pop a Xanax and sleep.” “I always talk about the little adjustments you have to make as your loved one gets worse. I’ve cried for about 50 mins and done the stupid rocking myself back and forth in efforts to comfort myself.” “I feel so powerless and I just want to make you healthy again. Every day I am so happy and put a smile on my face, trying to ignore the gnawing thoughts of your illness and how it is slowly destroying you from the inside.”
6	Family caregiving	“My only experience with this sort of thing is with my spouses family situation. With their family, they do not let the grandma go to her old house any more. I was told by my wife not to bring my mom back to her old home, as it would cause trouble or upset her like how her grandma got upset.” “My father-in-law was hiding from us just how bad it was (Not sure why as we where/are very active in their lives) and until FiL death she put on more of a front for us (didn’t always manage it, but managed enough to hide just how bad it was).” “My dad started answering the phone as my mom became more confused and often asked for her mom (died 50 years ago). Constant desire to go home and find her mom. Not recognizing my dad has been her caregiver through it all.”
7	Hospice care and decision-making	“She was released from the hospital into the care of one of her brothers, but as of today she has returned back home on her own. Basically, Family Care is not an option.” “He’s at the stage where he can opt for hospice care, but he’s choosing to prolong life treatment (or at least try.) I told him if he wants to do whatever he wants then hospice care is an option but he will live a lot less longer.” “After days of back-to-back seizures, she is in the hospital completely unresponsive and I got the news they don’t expect she will recover. I have an appointment tomorrow to go over our hospice care options. I thought we had more time. I wont ever hear her voice again. I used to get annoyed at all of the voicemails she would leave because I always called her back as soon as I could.”
8	AD/ADRD impact	“The husband has dementia and his wife was taking care of him. I could feel the heart break behind her words as her husband had clearly deteriorated over the years to resemble less and less of the person she described.” “She passed last night, a decade since it first became clear that the disease that had taken her sister had come for her as well. For most deaths ‘I’m sorry’ suffices, but we all know that with dementia that’s not exactly true.” “Over the past four years, my family has come to suspect that my mother has dementia, which doesn’t make a whole lot of sense, given that she’s 52. The further irony is that my grandmother has just now started displaying symptoms of dementia, at almost 90 years old.”
9	Hospital, assisted living, or memory care	“With my dad in the hospital, my mom has been a complete wreck! The doctor said she’d have to go into a home and she flipped out begging me to bring her to my house.” “My mother is in a memory care facility - she’s been there since last July. My father cant face the fact that this is a behavior situation, not a ‘see yet another doctor for a magic cream that will stop this.’” “I have been slowly priming my sister regarding getting mom ready for a home, as she is now truly requiring intense supervision for just about everything and i think my sister is realizing this as her decline has been incredibly apparent over the last few months since dad is gone.”

^a^AD/ADRD: Alzheimer disease and Alzheimer disease–related dementias.

### Qualitative Content Analysis

#### Overview

Through this analysis, we sought to situate prior NLP findings in the context of existing research on caregiving for people living with AD/ADRD. Specifically, we qualitatively analyzed a subset of our existing corpus, deductively mapping onto existing conceptual models of social support and AD/ADRD behavioral symptomology. We allowed for additional inductive themes to emerge.

#### Social Support

A total of 2 primary forms of social support were observed among Reddit users: informational and emotional. Of note, companionship behaviors and relatedness (other theoretical forms of social support) were not commonly observed in our data. Among the subcategories of informational forms of social support identified by Yao et al [47], 2 subcategories were observed frequently among our data: advice and teaching. Users sought advice from others related to a broad variety of topics, including help with decision-making (eg, seeking professional care support, admission to a care facility, and beginning hospice care); managing challenging symptomology (eg, aggression and wandering); coordinating care networks; and strategies to prevent burnout. For example, one user posted as follows:

I need help. With anger. With boundaries. Active things I already do: meditation, avoiding most substances, gardening, painting, journaling... tried therapy a few times they always offer meds which I dont [sic] want due to chronic illness I dont [sic] want to flare up... thank you.

Beyond this more generalized advice seeking, users sometimes sought or provided specific forms of teaching informational support. Often, these threads surrounded discussions of new medications or clinical trials for people living with dementia. Others offered practical tips and supports. For example, one user described the placement of an “orientation board” in a conspicuous place to provide useful information for their care recipient, including the day or date, expected appointments, weather, and other basics that helped reduce confusion.

In addition, we observed users both seeking and providing emotional social support. We observed a broad array of emotional support, including words of affirmation, expressions of care or concern, encouragement, sympathy, and understanding. Frequently, users expressed a vague or nonspecific need for support—that they had come to the forum seeking others with similar experiences who understood the challenges associated with caregiving. However, many users noted the sense of community and the value of the group’s shared experience in helping them individually. For example, a user thanked the group, saying the following:

I appreciate everyone so much. I know that what were [sic] experiencing with loved ones and or friends is painful, tiring, and other things. Your questions and experiences have given me a heads up on situations.

In a different case, users specifically coalesced and supported a member who was going through a particularly challenging period, including providing donations and financial support. The user thanked the group, saying the following:

Feeling a little better today and am very overwhelmed by the support. You all really did make me feel less alone and I appreciate your support and offers to talk when you could have easily scrolled past. I also appreciate the awards but next time, take that money and please donate it to the Alz [sic] association. I do love the hug and silver award though, that is very very very kind.

#### AD/ADRD Behavioral Symptomology

##### Overview

We coded AD/ADRD behavioral symptoms using the standard Neuropsychiatric Inventory [48]. We found users regularly discussed symptomology to approximate or assess disease progression, particularly in the context of major decision-making (eg, care levels and power of attorney). A total of 3 categories of neuropsychiatric behavioral symptoms were commonly observed in Reddit posts.

##### Agitation or Aggression

One primary behavioral concern that appeared consistently in the discussion was increasing levels of aggression or agitation. Users noted that as part of the disease progression, care recipients increasingly struggled with emotional regulation; this often led to outbursts and physical aggression. Users recalled incidents where care recipients had yelled at caregivers, thrown objects, and attempted physical violence—sometimes without explanation. For example, one user described attempts to keep a loved one from wandering by securing doors and fences on the property; during the resulting “tirade,” the care recipient “grabbed my brother by the front of his shirt and began screaming in his face” and they also noted the following:

He has threatened to slash the tires on the car. The other day I caught him just before he swung a metal pipe at the windshield of my rental car.

Among Reddit users, we found these discussions were often related to larger caregiving decisions. For some, the emergence of violence was an inflection point in seeking full-time or professional care. However, others sought help, as the aggressive behaviors of care recipients caused them to be removed from care facilities.

##### Delusions and Hallucinations

Another prominent theme in the data was increasing delusions among care recipients. These delusions often manifested as care recipients’ mistrust of those around them due to unfounded beliefs that these individuals would harm them in some way (eg, physical violence and theft) or as a conspiracy against them. Episodes of delusion covered a broad range of experiences, from a more benign suspicion that others were lying to more elaborate, reoccurring fantasies. For example, one user posted regarding regular hallucinations their parent had about a fantasy world:

[A] fantasy world he created involving red-robed people who came into the room uninvited and stayed overnight.

Regarding aggressive behaviors, many discussions around delusions and hallucinations were part of larger threads seeking support and understanding about dementia progression. Delusions were regularly discussed as a motivation for seeking external care support.

##### Nighttime Behaviors

The final commonly discussed behavioral symptom category related to nighttime issues. In the context of dementia, this category relates to a broad set of behaviors, including rising too early in the morning, awakening during the night, and excessive daytime napping—all generally related to sleep disturbance of some kind. Users regularly discussed the progression of sundowning—wherein individuals may experience restlessness, confusion, or irritability in the evening as daylight fades and progresses into the night. Users noted that sundowning symptoms continued into nighttime and often caused an inability to sleep or that patients would experience nightmares or terrors and stay awake throughout the night. In some cases, this inability to sleep resulted in the individual becoming fearful, seeking to escape, or taking additional medication. For example, one user noted the following about their mother:

[K]eeps getting up in the middle of the night, walking unassisted, digging around in her med suitcase taking God knows how many extra meds in the middle of the night.

sic

Commonly, these nighttime behaviors also had negative health repercussions for caregivers and family members who could not sleep themselves, either due to disturbances caused by the care recipient or by the need to provide supervision and care while the individual was awake.

#### Emergent Codes

##### Caregiver Anxiety or Guilt

The most frequently observed code across the entire data set related to caregiver guilt or anxiety, with users expressing distress related to their caring role and responsibilities, as well as their own emotions and personal challenges. For example, some users expressed fear of failing in their caregiving role, such as a user who expressed, “My biggest fear is that I’m not doing enough,” or another who said the following:

I’m struggling hard tonight. I want to want to fix this for my dad so badly but I can’t and I hate it. I hate that he has no idea what’s going on or why its happening and I hate that I can’t just make it all better for him.

sic

Another primary form of guilt and anxiety that emerged related to the users’ emotions and feelings. Some felt anger toward their care recipient. For example, some users noted they experienced emotional exhaustion related to caregiving and challenges coping with the loss of a loved one. One user, for example, lamented the loss of their mother’s cognitive function and was struggling with the decision to place her in an assisted living facility. The user experienced guilt around their feelings, noting the following:

[T]o me, my mother has been gone a long time. Am I an asshole for not wanting to see her...?

sic

Furthermore, multiple users expressed guilt associated with feelings of relief (both realized and expected) upon the passing of the person in their care, such as the user who stated the following:

I feel sad but also I feel free...I think sometimes “now I can start my life” and then I feel selfish and sad.

##### Decisions-Making About Care Facilities

Another commonly observed theme in the data involved decision-making about care facilities and other forms of assisted living. Prospectively, users sought advice and validation related to deciding when in the disease progression it was appropriate to place their loved ones in an assisted living facility. In addition, users sought practical advice on finding and selecting the right place and evaluating quality of care. However, many posts discussed challenges related to patients already in an assisted living facility. For some, there were unexpected or short-notice care expectations, even after the individual was placed in an assisted living facility. One user noted as follows:

The facility staff are telling me we either need to coordinate a sitter to literally sit outside his door 24/7 and physically keep him from leaving, or to send him to a mental institution until they get his meds right. This change needs to happen within 24 hours.

Other users were disappointed with the quality of care their loved ones were receiving. For example, one user said the following:

She was able to get out via an alarmed door, which apparently didn’t sound, get past reception, out the front door and walked up a super busy street to a grocery store 1/2 mile away! The street is almost a highway with. 3 lanes of traffic on each side. I don’t know all of the details yet but clearly this is not acceptable...I’m so angry that this could happen. My family is paying a ton of money to keep them safe and this happens.

Therefore, the myriad challenges associated with care facilities were common themes in the discussion on dementia-related subreddits.

##### Legal and Financial Planning

Users also commonly discussed the challenges associated with legal and financial planning for their loved ones living with AD/ADRD. These posts largely centered on the high financial costs of professional care and assisted living facilities and the steps caregivers and families could take to afford this support and protect family assets. Many users noted that their loved ones did not have sufficient resources (eg, retirement savings, long-term care insurance, and state support) to cover the costs of needed full-time care. In some cases, users reported subsidizing their loved ones’ care with their own funds:

I used to be what I would consider reasonably well off, now I have nothing left and struggle daily to make ends meet.

Others described an impossible tension, wherein they could neither afford to pay for needed care nor quit their own jobs to provide full-time care, such as the user who said the following:

My only word of advice is, unless you’re a millionaire and can afford home care, which we were most certainly not, there is no solution and no easy road to this disease.

One common concern in the discussion of care was the protection or leveraged use of assets, specifically houses owned by the care recipient. For example, one user described a situation in which their parent could not live in his home because he needed rental income to pay for his care, effectively forcing him to move in with relatives. Others noted that owning a home precluded access to social support programs as it counted as a substantial asset that had to be depleted before receiving assistance. Several threads related to strategies for protecting such assets (eg, placing the home in a trust) or challenges in liquidating them (eg, relatives living in the house or unwillingness to sell). Furthermore, these conversations commonly discussed the need to get legal affairs in order, such as establishing power of attorney and writing a living will early in the disease progression.

### Thematic Comparison

Across both forms of data analysis, we observed similar overall topical structures within Reddit posts related to AD/ADRD caregiving. The most salient NLP-identified topics were very general and focused on making inquiries or sharing emotions with the forum. In our qualitative analysis, the most common coding was quite similar, related to users’ expressions of emotion. However, through qualitative coding, we identified added nuance to these emotions of guilt, anxiety, and the struggles of AD/ADRD caregiving. Furthermore, we noted users sometimes expressed feelings that may be socially unacceptable outside an anonymous online space. For example, some users expressed relief upon the death of their care recipient. While likely not an uncommon emotional response given the myriad challenges associated with care, this relief may be uncomfortable to express to others for fear of judgment.

Similarly, the corpus-wide NLP analysis identified information seeking as a primary use of Reddit forums for AD/ADRD caregivers. However, in our qualitative coding, we identified specific informational needs of caregivers (eg, legal and financial planning advice) that were not apparent in the wider analysis. Moreover, we found through the qualitative analysis that users often sought normative standards for decision-making at common key time points in the disease progression, such as the “right” time to move a care recipient to professional or end-of-life care. While we observed themes related to assisted living and hospice care in the NLP, the qualitative coding provided a deeper understanding, finding that users were most often seeking external standards from others, which they could then apply to their unique situations and reduce doubt in their own decision-making. Therefore, our broad NLP analyses gave a high-level, atheoretical overview of themes in a large corpus beyond what is generally feasible for manual, human-driven analysis. Our qualitative coding fleshed out these findings, incorporating existing conceptual frameworks to contextualize and provide a more detailed understanding of the social support and information-seeking behaviors of AD/ADRD caregivers on Reddit.

## Discussion

### Principal Findings

Users sought community and informational support in dealing with the challenges associated with caregiving for people living with AD/ADRD. Consistent with prior literature, users reported a variety of emotional challenges related to providing care [34-36]; caregiver guilt and anxiety was a highly salient theme across the forum. The sources and manifestations of these emotions were highly variable across the forum. For example, users noted feelings of inadequacy in their capacity to care for loved ones, guilt associated with a lack of patience for their care recipient, loneliness and social isolation, burnout, and anxiety related to the varied stressors associated with care. While caregiver burden is well-established in the literature [15], it was particularly salient in the online space, wherein this seemed to be a primary point of conversation between users. Given the documented social stigmatization of dementia caregivers [37], users may seek to express these feelings and find support online as they do not feel comfortable expressing their challenges in day-to-day life. Furthermore, and unique to the anonymous online space, users felt empowered to discuss potentially taboo topics, including relief upon the passing of their care recipient. It is important to note the subreddits analyzed in this study are moderated to avoid bullying and spam and to establish a respectful dialogue in the forum. Notably, users may feel particularly safe to express these otherwise stigmatized beliefs and challenges. Creating this trust and safe space to discuss the complex emotions associated with dementia care may be similarly useful in face-to-face caregiver support interventions.

Consistent with other studies, Reddit users discussed myriad behavioral symptoms of AD/ADRD and strategies for providing effective care related to each symptom [19,20]. Manual coding identified instances of 14 different behavioral symptoms of dementia. The most identified among these were agitation or aggression, delusions, and bathroom or toileting issues. These are, perhaps, not surprising, given that these are symptoms with higher associated costs or challenges of care. Therefore, these may be the symptoms most discussed when seeking support. Often, users reported multiple symptoms together in the same post, consistent with the nonlinear nature of disease progression. Many users sought to map symptomology onto a disease progression timeline to better understand their own situation. However, as was noted by multiple users, this is challenging as dementia can present and progress differently across individuals. As such, the presentation of behavioral symptomology was often highly variable across the sample.

Finally, discussions with the forum also often related to difficult decisions facing caregivers of people living with dementia [4,49]. Many posts focused, for example, on the legal and financial planning challenges of care; users provided resources to others to help create power of attorney documentation and to navigate health care systems. The complicated legal and financial challenges associated with dementia and end-of-life care have been documented elsewhere. In our sample, users sought to crowdsource resources to navigate these complex systems, potentially without the high costs associated with hiring specialist attorneys and personal financial planners. Furthermore, users sought feedback associated with care decisions, particularly the proper timing for moving their loved ones into a full-time care facility. User discussions often compared symptomology and family circumstances, seeking validation or support in making the choice to seek professional care help. Again, these discussions often centered on stigma, fears associated with care quality, and the financial costs of professional care. As users faced difficult but common decisions related to transitions in care—times associated with higher perceived caregiver burden—they sought online emotional and informational support.

### Implications for Practice

Our study findings suggest the need for increased social support structures for AD/ADRD caregivers, a special population who may experience challenges associated with seeking information and other forms of support on the web. This is consistent with the wider literature surrounding caregiver burden, suggesting high rates of burnout and needs for additional support beyond localized networks [34,35]. However, our study findings introduce important additional considerations. Recent literature has begun to document mental health risks associated with extensive social media use, particularly linked to unfavorable social comparison (ie, to others with fewer perceived challenges) [50]. There are further risks associated with seeking health-related information on the internet, given the high rates of misinformation found on social media [51]. Therefore, despite the somewhat closed and moderated nature of dementia-related subreddit forums, the overall absolute value for AD/ADRD caregivers of seeking support and information therein is unknown. Online resources and spaces for social connection, such as Reddit, which also feature expert fact-checking and seek to reduce harmful social comparisons, may be needed to support caregivers. Furthermore, there are opportunities for the development of mobile apps specific to AD/ADRD caregiving; however, additional research on the relative benefit of new mobile apps versus commonly used social media platforms is needed [40].

In addition, users noted specific informational needs with which they struggled to find high-quality support, including legal or financial planning and strategies for coping with feelings of inadequacy or guilt. These gaps in readily available information offer opportunities for intervention. For example, programs designed to teach caregivers about complex legal and financial planning documents (eg, power of attorney, living wills, and advance directives) may reduce burden. Given the well-documented financial strain associated with providing care, a centralized, online information hub for legal and financial planning may increase the capacity to proactively manage the complex tasks associated with estate planning and long-term care financing. Furthermore, specialized support groups for caregivers may use acceptance and commitment therapy techniques, which emphasize self-compassion around perceived personal shortcomings [52]. Acceptance and commitment therapy techniques are increasingly used in family caregiving settings (including for dementia), with promising early acceptance or feasibility and results related to psychological flexibility, which is the capacity to stay in contact with the present, irrespective of negative thoughts or feelings [52-54]. Unfortunately, access to such interventions and therapies remains limited, leading caregivers to seek information and social support in free and open online spaces. Future intervention work may leverage such opportunities to create readily accessible supports to assist caregivers in managing both specific care tasks (eg, financial management) and emotional challenges associated with caring for a loved one living with AD/ADRD.

### Limitations and Future Directions

As with all research, this study has certain limitations that should be considered. First, the sample was drawn from a single, moderated, online space, which may limit some of the most extreme responses from being published. Reddit users are, on average younger, highly educated, and more likely to be male than the general population. Furthermore, because data were published anonymously and are self-reported, we have no mechanism by which to directly assess truthfulness. However, this anonymity was a key feature to our findings and has been reported as a strength in other social media studies. Future research may explore AD/ADRD caregivers’ use of different social media platforms, as social media use varies across demographic groups. Similarly, future research may examine differences between social media platforms, due to the differing nature of the content (ie, images, text, and video) found on each platform. In addition, while we analyzed a large sample using computational methods, the qualitative process for deeper analysis was limited to a smaller subsample. Therefore, our findings are not necessarily representative of all dementia caregivers and should be considered with these limitations in mind.

### Conclusions

In this study, we used both computational and traditional qualitative analyses to explore the experiences of caregivers of people living with AD/ADRD who posted to the social media channel Reddit. Using an unsupervised topic modeling approach, we generated a 10-topic solution (coherence score=0.38) from a corpus of more than 6500 posts. These topics broadly centered on emotional and logistical challenges associated with care, as well as a cluster of topics associated with various symptomology. To gain a deeper understanding of these topics, we conducted a qualitative review of a subset of posts. Users noted high levels of burden, guilt, and anxiety associated with caregiving. Users sought emotional and informational support to manage the behavioral symptomology of their care recipients and to make key legal, financial, and other care-related decisions. Interestingly, the anonymous and moderated nature of the Reddit platform seemed to reduce the perceived risk of stigmatization, allowing users to express difficult and complex emotions related to the care experience, including resentment and relief upon the passing of a loved one. These findings suggest the need for additional caregiver support interventions to reduce burden and improve overall well-being for both the caregiver and care recipient.

## Abbreviations

AD/ADRD: Alzheimer disease and Alzheimer disease–related dementias
BERT: Bidirectional Encoder Representations from Transformers
LDA: latent Dirichlet allocation
NLP: natural language processing
